# Supplementary material for: Far-field nanoscale infrared spectroscopy of vibrational fingerprints of molecules with graphene plasmons
Source: Nat Commun. 2016 Jul 27;7:12334. doi: 10.1038/ncomms12334 (PMC4974468; doi:10.1038/ncomms12334)
Supplement: Supplementary Information — Supplementary Figures 1-15, Supplementary Notes 1-4, Supplementary References [file ncomms12334-s1.pdf]

Supplementary Figures

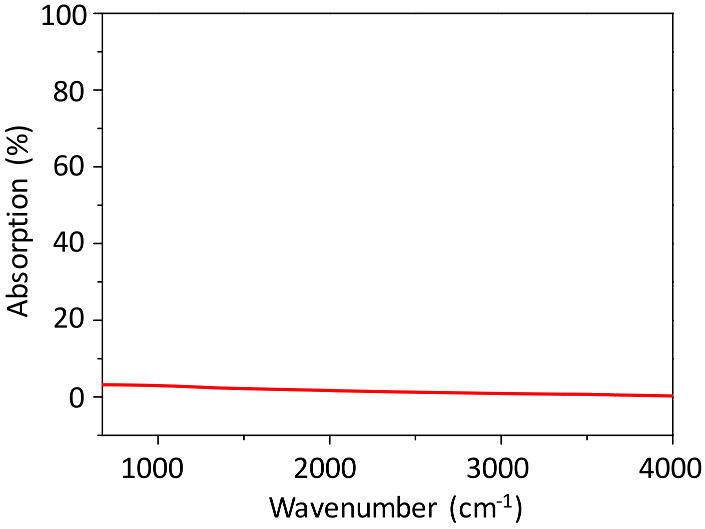

Supplementary Figure 1. Absorption spectrum of a 300 nm CaF_2 film on silicon. It is highly transparent in the range from 675 to 4000 cm^{-1}, with no distinctive resonant mode in this spectrum.

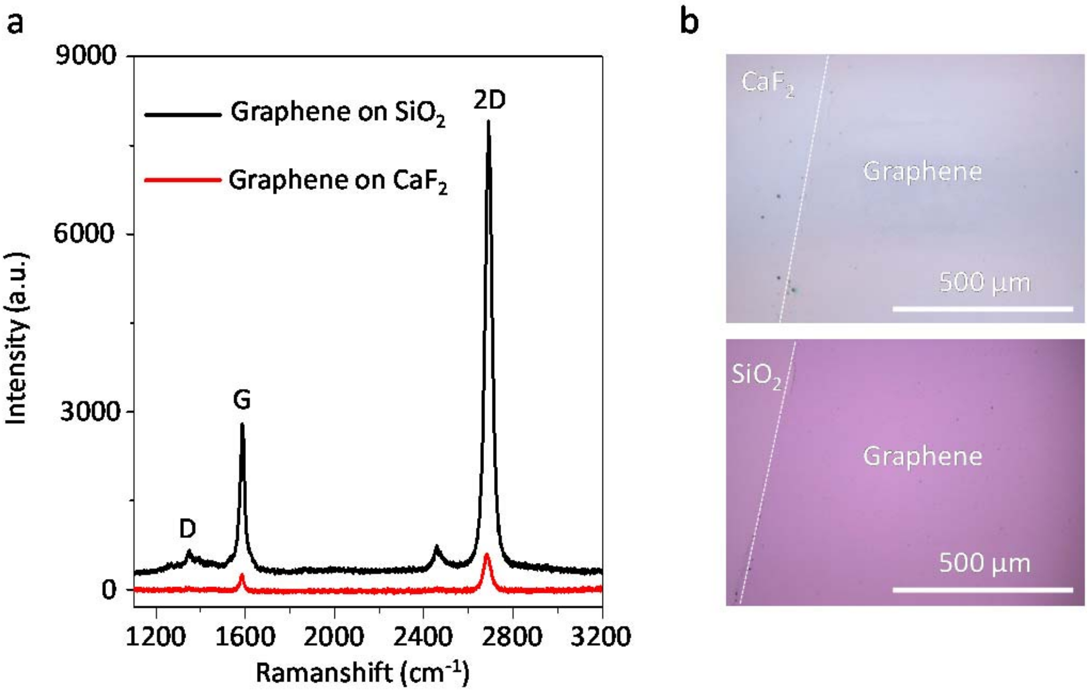

Supplementary Figure 2. (a) Raman spectra of monolayer graphene transferred on SiO_2 (black curve) and CaF_2 (red curve) substrates. (b) Optical micrographs of the graphene sheet on CaF_2 (top) and SiO_2 (bottom) substrates.

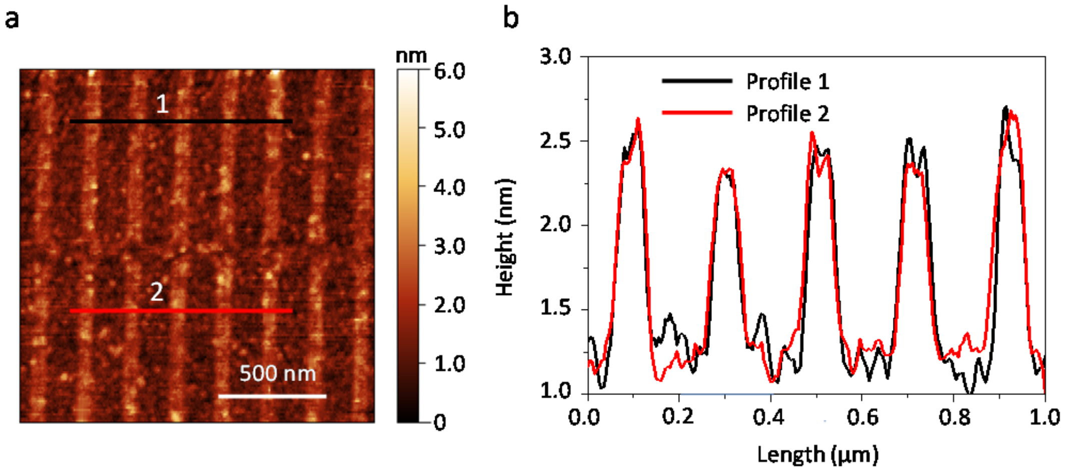

Supplementary Figure 3. (a) AFM image of graphene nanoribbon array. Ribbon width (*W*): 80 nm; Width-to-pitch ratio: 1:3. (b) Line-scan profile of the image shown in (a), corresponding to the black and red lines, respectively. The graphene nanoribbons have a uniform thickness of 1.5 nm.

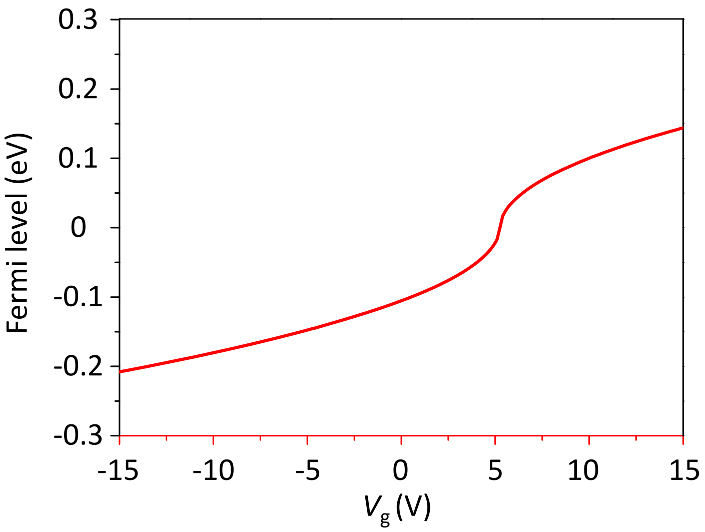

Supplementary Figure 4. The dependence of graphene Fermi level on V_g.

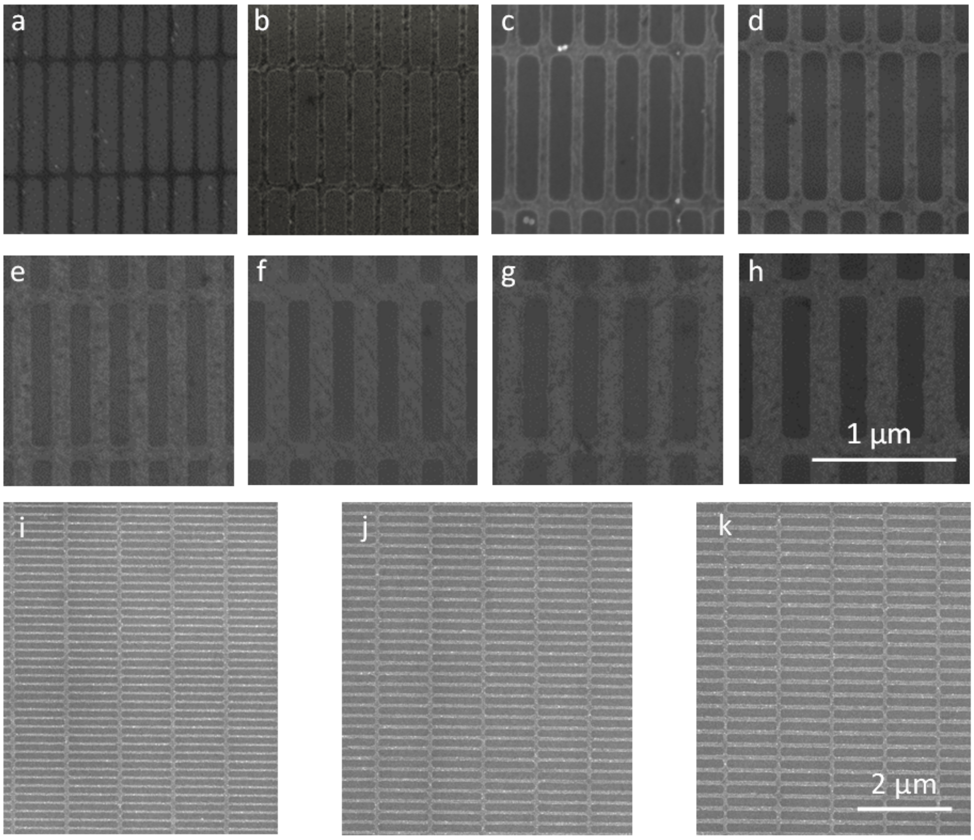

Supplementary Figure 5. SEM images of graphene nanoribbon arrays with different ribbon width on SiO_2 substrates (a-h) and CaF_2 substrates (i-k).

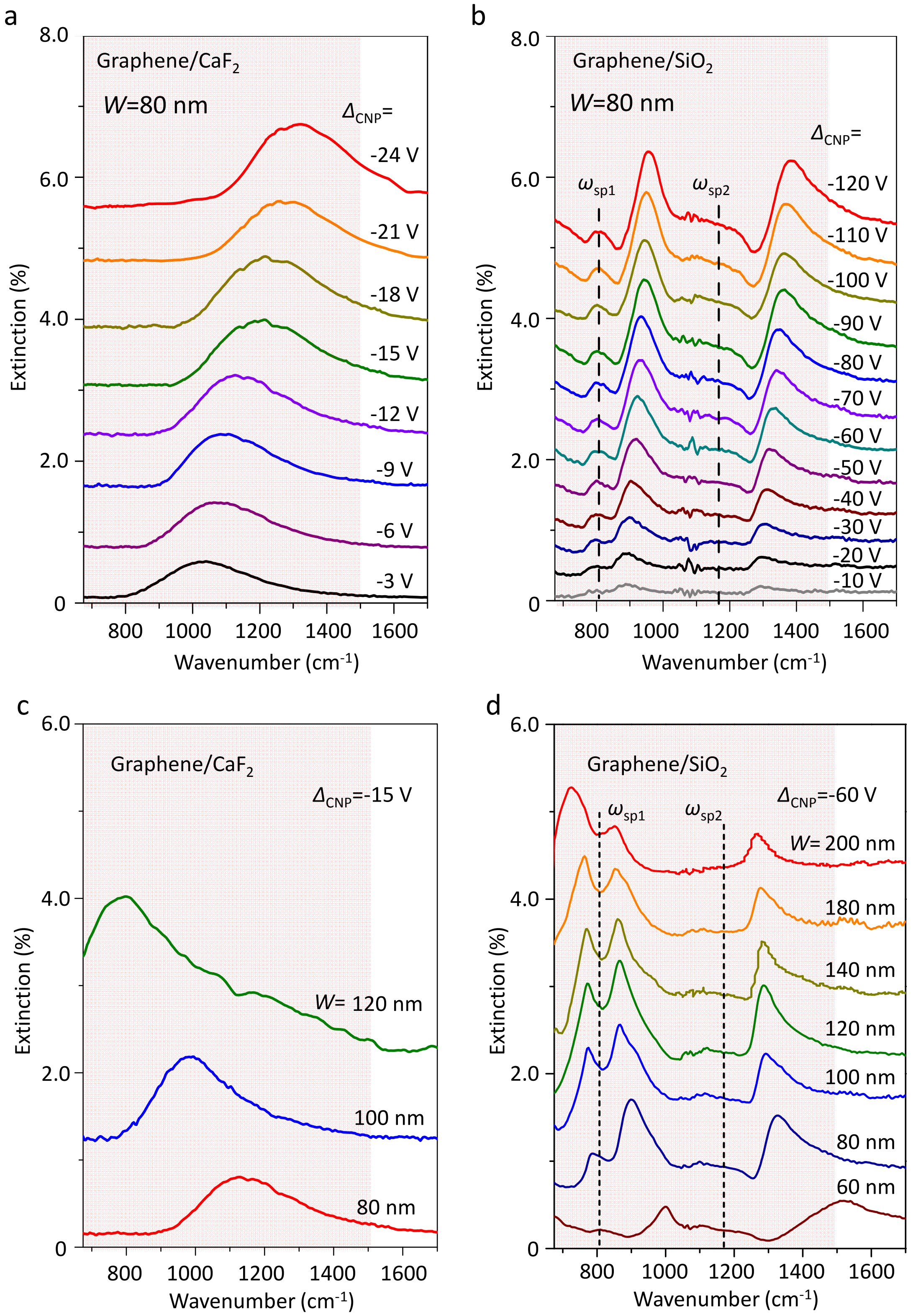

Supplementary Figure 6. Extinction spectra of graphene nanoribbon arrays at varied Fermi levels on CaF_2 substrate (a) and SiO_2 substrate (b). Δ_{CNP} means the applied gate voltage shifted from the charge neutral point voltage which determines graphene Fermi levels. Extinction spectra of the graphene nanoribbon arrays with varied ribbon width (*W*) on CaF_2

substrate (c) and SiO_2 substrate (d). Graphene plasmon couples strongly with the two surface optical (SO) phonons of SiO_2 at 806 cm^{-1} (ω_{sp1}) and 1168 cm^{-1} (ω_{sp2}), indicated by vertical dashed lines in (b) and (d). This strong plasmon-phonon coupling induced electromagnetic filed cancel between plasmon and phonons, and shaped the wide plasmon resonance peaks (as shown in b and d) into well separated three sharp peaks, which cannot even cover half of the fingerprint region (red shadow). While on our CaF_2 substrate, the graphene plasmon resonance can be continuously tuned electrically in a wide spectrum range without suffering any hybridization effects from phonons. In addition, Graphene plasmon frequency changes with ribbon width accordingly following the scaling behavior of $W^{-1/2}$. [1-3] By varying the width of graphene nanoribbons, graphene plasmon resonance frequency can be regulated to cover the whole fingerprint region, and even the mid- and far- infrared region, which broadens the detectable spectrum range of the graphene plasmon-based IR sensor to a very large extent. In all figures, red shadow indicates the vibrational fingerprint region.

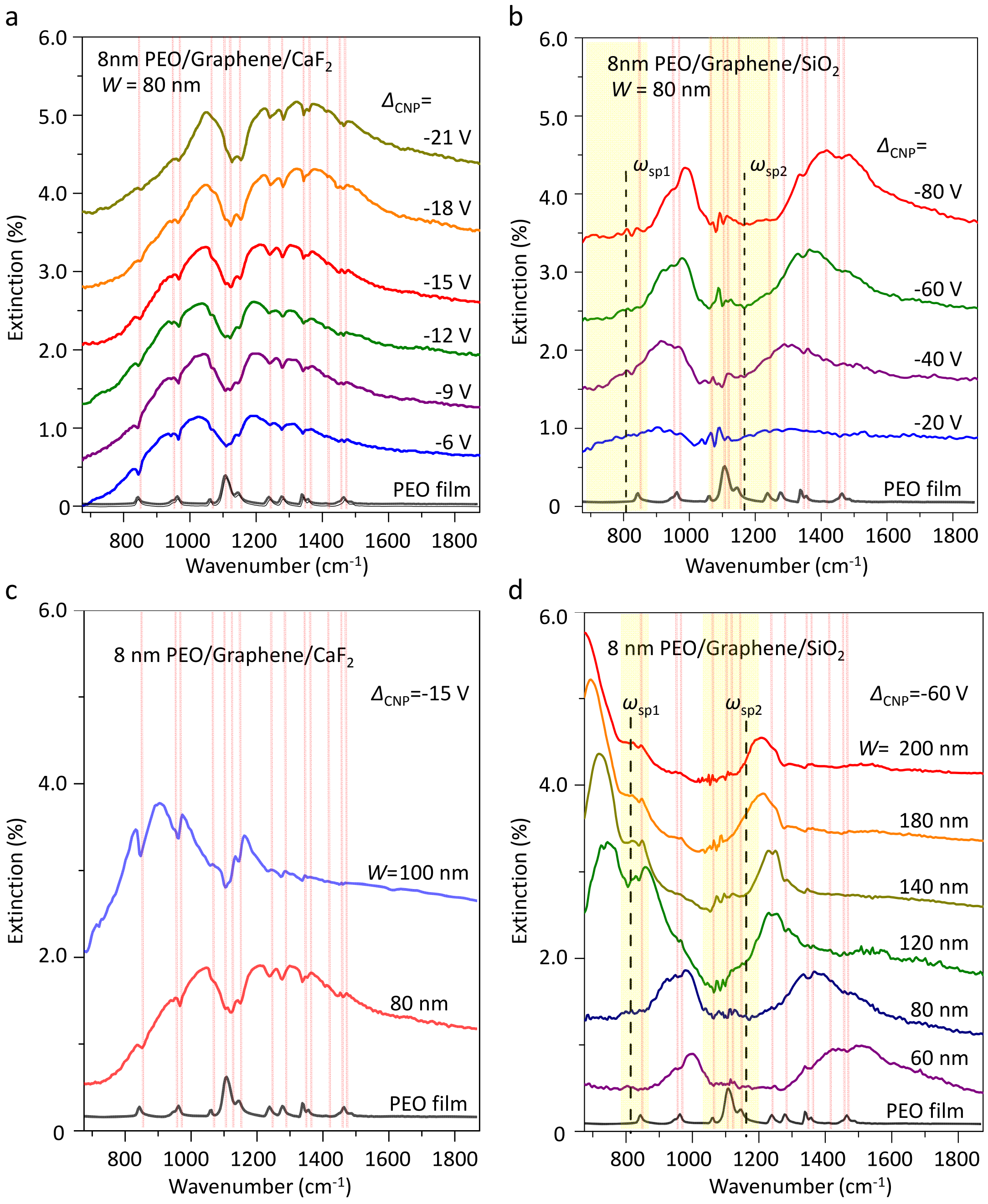

Supplementary Figure 7. Extinction spectra of graphene nanoribbon arrays after coating with 8 nm PEO film on CaF_2 substrate (a, c) and SiO_2 substrate (b, d). By varying the Fermi level (a, b) and the width (c, d) of graphene nanoribbons, graphene plasmon resonance frequency can be regulated to cover the whole fingerprint region. As shown, all the vibrational modes of the analyte 8 nm PEO film in the fingerprint region can be enhanced and detected by the undisturbed graphene plasmon on our CaF_2 substrate (a, c). However, the phonon-induced transparency in the hybrid peaks of graphene plasmon on the SiO_2 substrate (b, d) are very weak. It is because that the strong coupling between the graphene plasmons and substrate phonons confines the electromagnetic energy between the graphene and substrate and results in very low near-field enhancement on the top graphene surface for sensor applications. Especially in the yellow shadow regions which marks the anticrossing

regions of the hybrid peaks resulting from plasmon-phonon coupling, there even no enhancement signal for analyte. The grey line at the bottom is an absorption spectrum of 430 nm PEO used to mark the PEO vibrational modes. The vibrational modes of PEO in the fingerprint region are indicated by red vertical lines. The two SO phonons of SiO_2 are indicated by vertical dashed lines in (b) and (d).

a

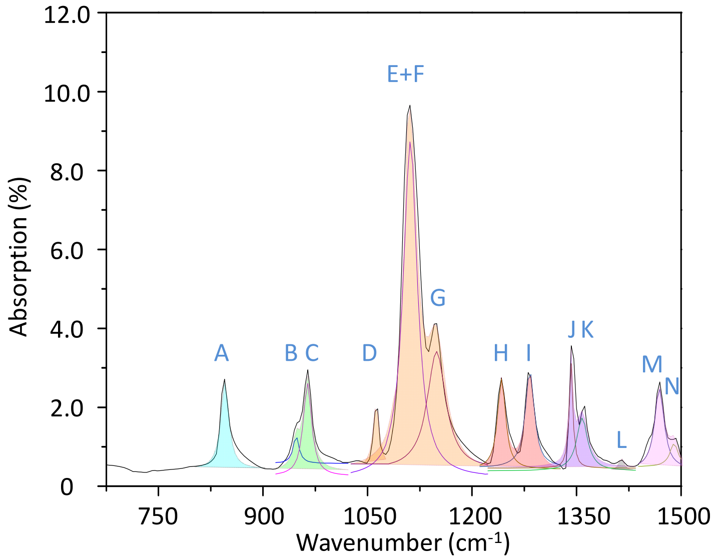

b

Peak	Area
A	39.89
B	9.12
C	44.02
D	16.43
E+F	273.13
G	117.77
H	43.34
I	51.78
J	25.86
K	35.47
L	2.88
M	34.54
N	10.41

Supplementary Figure 8. (a) Absorption spectrum of a ~430 nm thick PEO film. The Lorentz line shapes (the thin solid curves) are used to fit the IR absorption peaks of PEO molecules. The shaded areas with different colors indicate the superposition of the fitted peak areas. (b) Peak area of each vibrational modes indicated in (a).

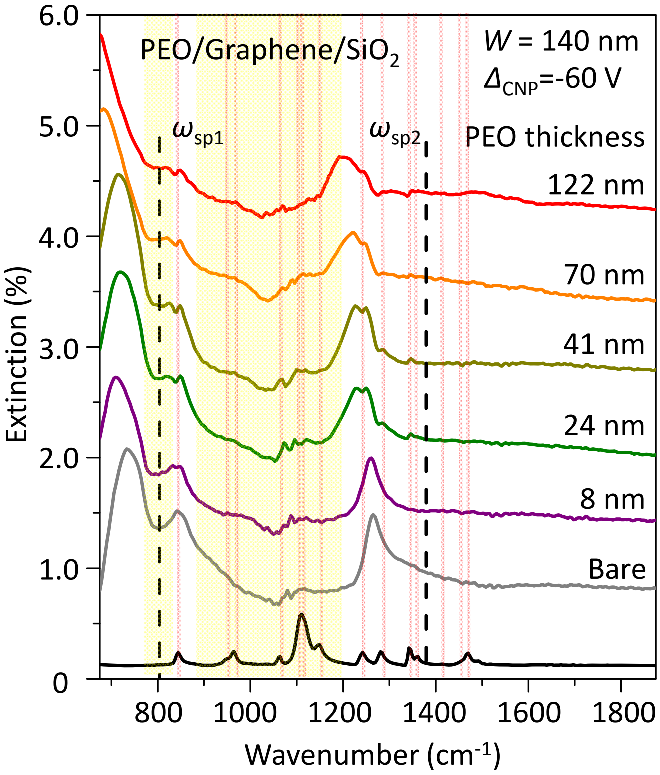

Supplementary Figure 9. Extinction spectra of the graphene nanoribbon arrays after coating with different thickness of PEO film on SiO_2 substrate. As shown, the phonon-induced transparency of PEO in the hybrid peaks of graphene plasmon on SiO_2 substrate are very weak. Especially in the yellow shadow regions, there are barely signs of enhancement signal for analyte. Even the thickness of PEO film increased, the enhancement signal, that is the depth of the phonon-induced transparency in the hybrid peaks, did not become stronger. The vibrational modes of PEO in the fingerprint region are indicated by red vertical lines. The two SO phonons of SiO_2 are indicated by vertical dashed lines. Yellow shadow regions marks the anticrossing regions of the hybrid peaks resulting from plasmon-phonon coupling.

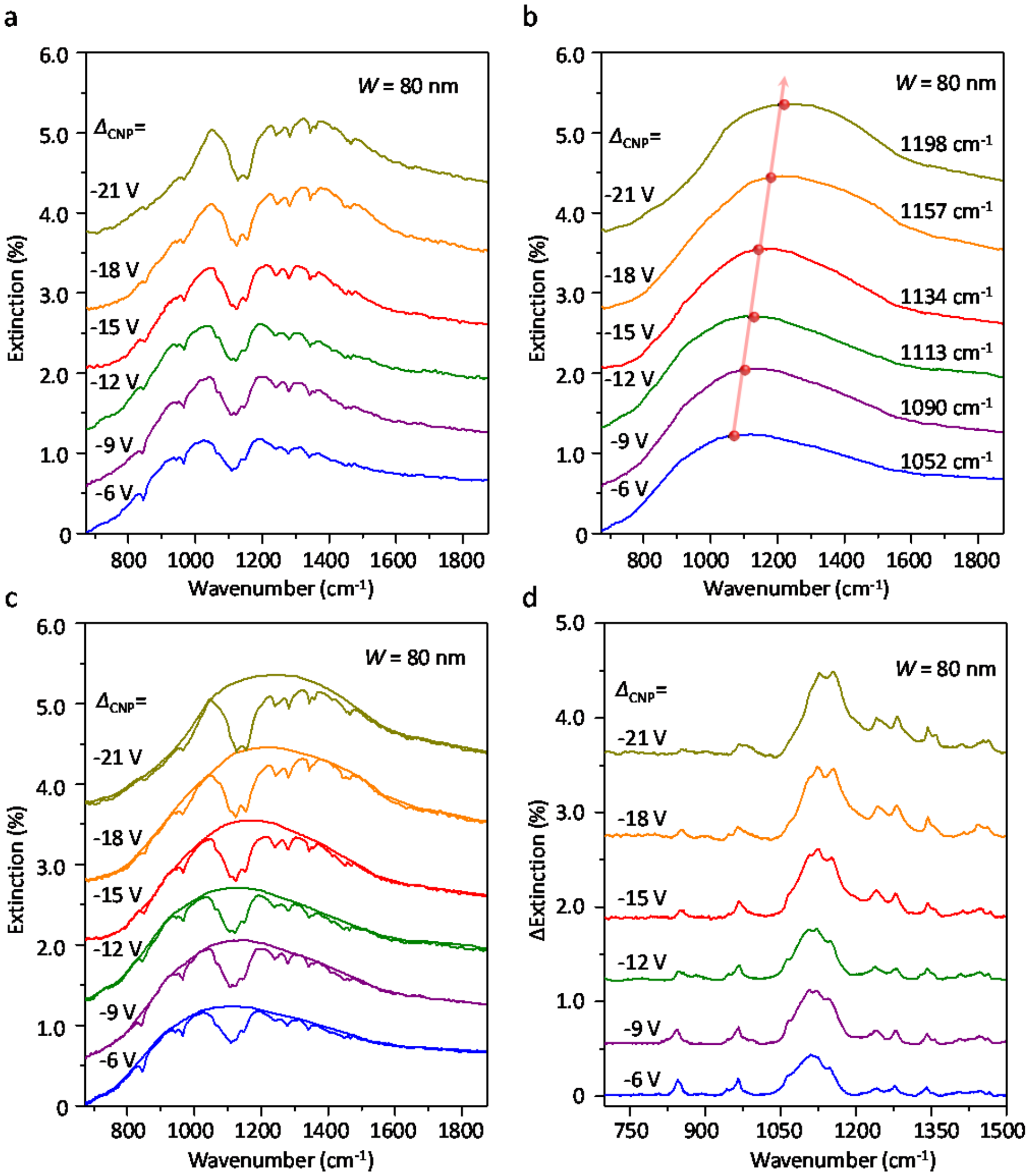

Supplementary Figure 10. (a) Extinction spectra of the same graphene nanoribbon array coated with an 8-nm PEO film at varied Δ_{CNP}; (b) Fitted features of graphene plasmon without coupling with phonons using the lineshapes derived from the pristine plasmonic resonance peaks. The red spots represent the plasmon resonance peaks; (c) Comparison between (a) and (b); (d) Extracted absorption feature of PEO vibrational modes after plasmonic enhancement.

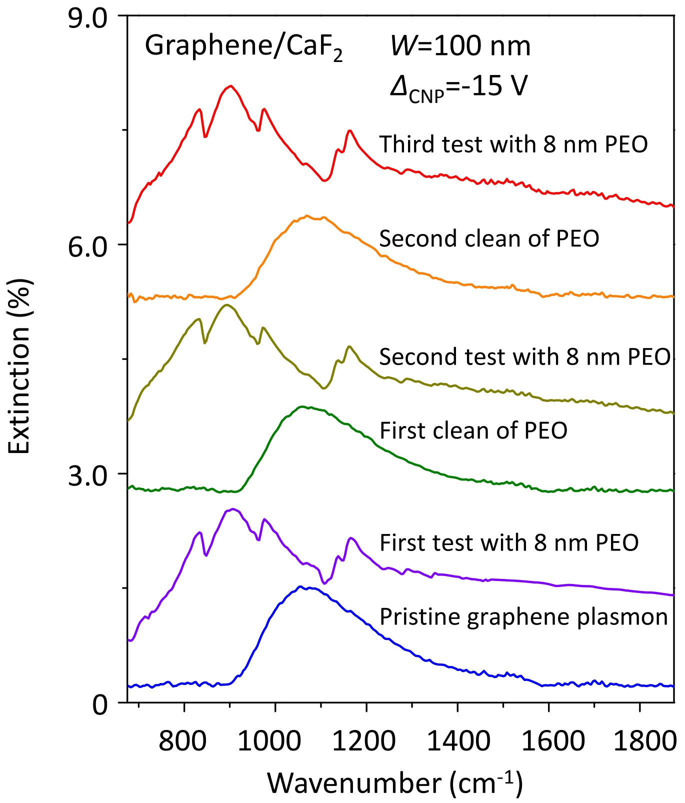

Supplementary Figure 11. The recycling of our graphene/CaF_2 sensors. When the 8 nm PEO was washed away, the graphene/CaF_2 plasmonic sensor maintained its property and had the same ability to detect the analyte. After several cycles, there is nearly no performance degradation for the sensors. It shows that our device indeed can be used repeatedly.

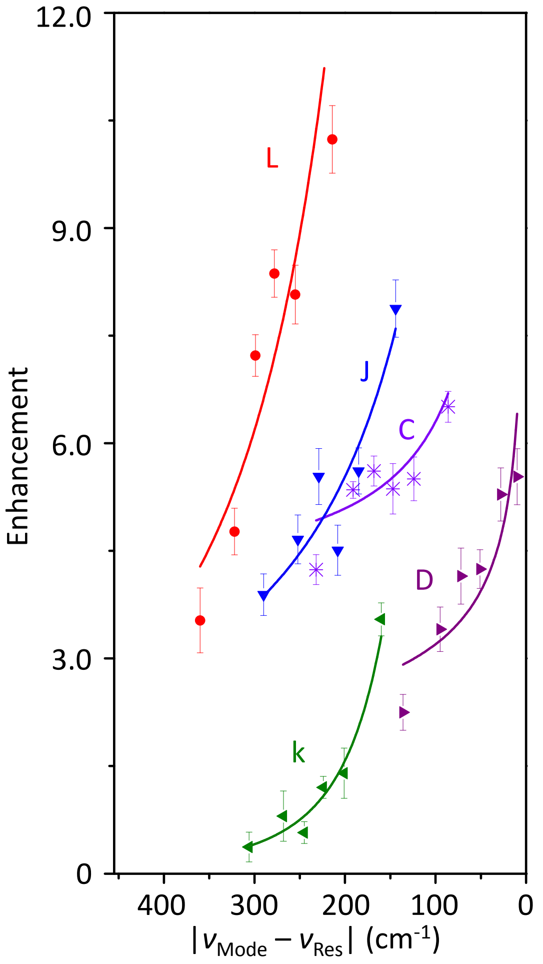

Supplementary Figure 12. The relationship between enhancement factor of vibration modes and their distance to the plasmon resonance frequency of the modes C, D, J, K and L. The error bars in the plots are standard deviation from large numbers of measurements.

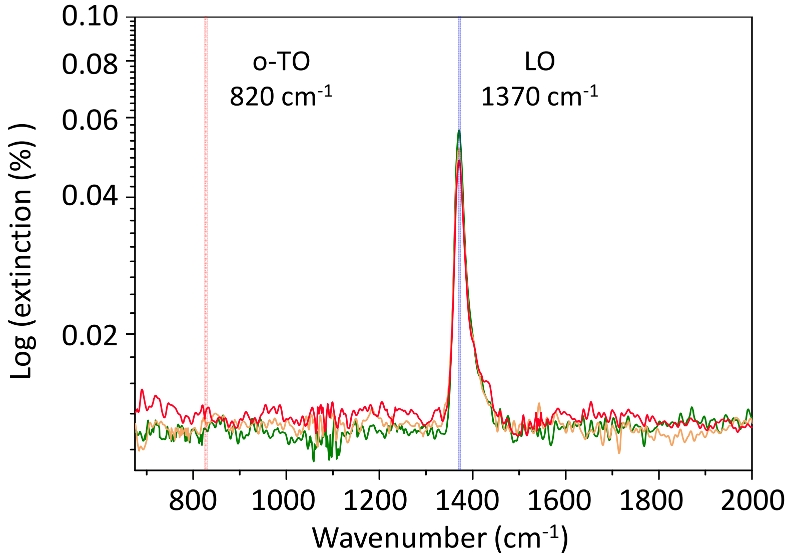

Supplementary Figure 13. Absorption spectra of three typical monolayer h-BN samples. The red and blue vertical lines indicate the positions of the out-of-plane (the transverse optical phonon mode at ~820 cm^{-1}, o-TO) and in-plane (the longitudinal optical phonon mode at ~1370 cm^{-1}, LO) modes in monolayer h-BN, respectively.

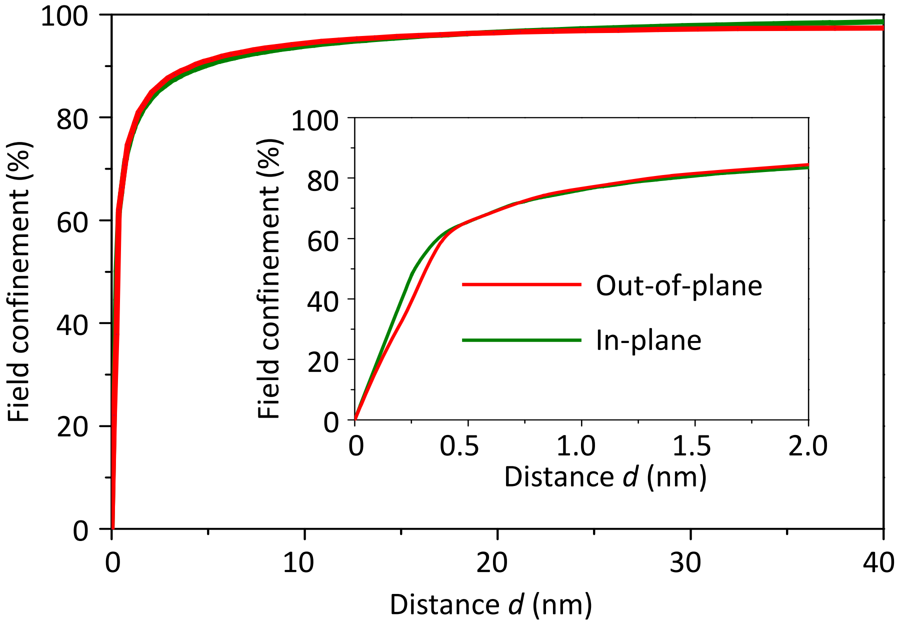

Supplementary Figure 14. Space-integrated near-field intensity confined within a volume extending a distance d along different directions, green curve represents out-plane direction (*z*) and red curve represents in-plane direction (*x*), respectively. Inset: A zoom-in image in the distance range from 0 to 2 nm.

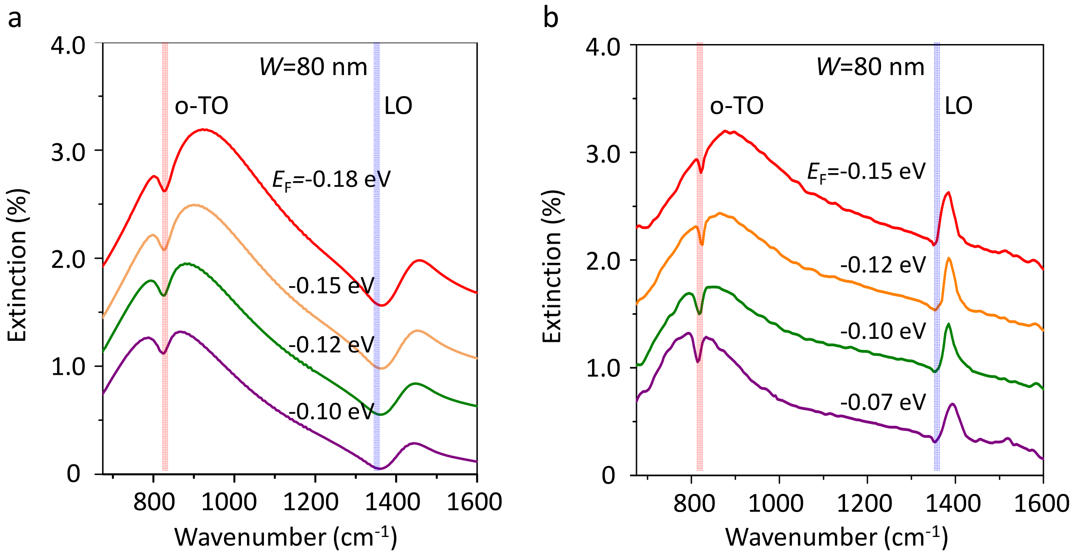

Supplementary Figure 15. Comparison of the plasmon-phonon coupling between the FDTD simulation results (a) and experimental results (b). In the theoretical model, the thickness of

monolayer h-BN film is 0.34 nm; the width of graphene nanoribbon is 80 nm. Surface conductivity of graphene was defined by Kubo formula in a complex form consisting of interband and intraband contributions [11]. The permittivity model of the h-BN film is obtained from reference 12.

Supplementary Note 1. The calculation of graphene Fermi levels

The calculation of carrier densities and Fermi levels of graphene on CaF_2 is as follows. From the determined transfer characteristics, capacitance and thickness of the dielectric, we calculated the carrier density of graphene using a standard parallel plate capacitor model. For CaF_2 thin film, using a relative dielectric constant of 6.8 and a thickness of 300 nm, the capacitance was calculated as 0.021 $\mu F cm^{-2}$. The dependence of the carrier density on the gate voltage satisfies the equation:

$$V_g - V_{CNP} = \frac{\hbar | \nu_F | \sqrt{\pi n}}{e} + \frac{ne}{C_g},$$

where V_g is the gate voltage, V_{CNP} is the charge neutrality point voltage, ν_F $=1.1\times10^6$ m s^{-1} is the Fermi velocity, n is the carrier density and C_g is the capacitance of gate dielectric. Combined with the relation between E_F and the carrier density of graphene $E_F = \hbar | \nu_F | \sqrt{\pi n}$, we obtained the relation between n , E_F and the gate voltages.

Supplementary Note 2. Method to extract PEO molecule vibrational signals from plasmon extinction spectrum and calculations of enhancement Factor

The extinction spectra of our experiments (Ex^{PM} , Supplementary Figure 10a) are originated from graphene plasmon coupled with PEO molecular vibrational modes.

This phonon-induced transparency has been understood as a special case of coherent destructive interference between phonon resonances and the plasmon polaritons.[4, 5] Therefore, we can obtain the absorption spectrum (Ex^{M}, Supplementary Figure 10d) after plasmonic enhancement by subtracting our extinction spectrum (Ex^{PM}, Supplementary Figure 10a) from the pristine graphene plasmon spectrum (Ex^{P}, Supplementary Figure 10b), following the method in Refs. [6,7] (that is, $Ex^{M}= Ex^{P}- Ex^{PM}$). Then, we decompose the absorption spectrum (Ex^{M}, Supplementary Figure 10d) after enhancement into peaks at different mode positions with a form of Lorentz function (as shown in Fig. 2c of the main text). After, we calculate the field enhancement factor at different modes (Fig. 2d in the main text and Supplementary Figure 11) by comparing the decomposed peak area (in Fig. 2c) with the absorption peak area of an 8-nm-thick PEO film without enhancement (deduced from a 430-nm thick PEO film, see Supplementary Figure 8 above).

Supplementary Note 3. Recycling experiments of the graphene/CaF_2 sensor

Supplementary Figure 11 shows that our device can be used for multiple times when analyte can be removed via certain methods such as washed away by solvent or evaporated in vacuum over elevated temperature. After several cycles, there is nearly no performance degradation for the sensors. It shows that our device indeed can be used repeatedly.

Supplementary Note 4. The coupling mechanism of monolayer h-BN o-TO mode and graphene plasmons

Here, we explain the possible coupling between the o-TO mode and the graphene plasmon by a classic electromagnetic coupling theory as follows:

Monolayer h-BN used in our experiments has only three optical phonons due to its simple atomic structure: LO, o-TO and in-plane (i-TO) phonons. The atom vibration directions of LO ($\mathbf{e}_L$), i-TO ($\mathbf{e}_{iT}$) and o-TO ($\mathbf{e}_{oT}$) are along x, y and z direction as shown in Fig. 3b of the main text, where graphene nanoribbon is along the y direction and the wave vector of graphene plasmons is along the x direction. For each λ∈ mode (LO, i-TO and o-TO), the displacement between the two atoms in a unit cell can be written as $u_{\lambda l} = u_{\lambda q}\exp(i\mathbf{q}.\mathbf{R}_l)\mathbf{e}_\lambda$, where $\mathbf{R}_l$ specifies a unit cell and $u_{\lambda q}$ is spatially homogeneousness of λ phonon. This relative lattice displacement introduces a dipole $en_B^\lambda u_{\lambda q}\mathbf{e}_\lambda\exp(i\mathbf{q}.\mathbf{R}_l)$, where n_B^λ is the effective Born charge of the boron ions in the unit of the fundamental charge e, $n_B^{LO} = n_B^{i\text{-TO}} = 0.60$, $n_B^{o-\text{TO}} = 0.15$ (ref. 8).

In the long wavelength limit, the polarization field of the optical phonon λ generates an electric potential (Φ_λ), which is given at a position of $\mathbf{r} = x\mathbf{e}_L + y\mathbf{e}_{iT} + z\mathbf{e}_{oT} (z>0)$ as follows:

$$\Phi_\lambda(\mathbf{r}) = \sum_l \frac{en_B^\lambda u_{\lambda q}\mathbf{e}_\lambda.\mathbf{r}_l}{4\pi\varepsilon_0 r_l^3}\exp(i\mathbf{q}.\mathbf{R}_l),\ \mathbf{r}_l = \mathbf{r} - \mathbf{R}_l. \tag{1}$$

The summation over a lattice vector $\mathbf{R}_l$ translates into the following integral,

$$\begin{Bmatrix}\Phi_L(\mathbf{r})\\ \Phi_{oT}(\mathbf{r})\\ \Phi_{iT}(\mathbf{r})\end{Bmatrix} = \frac{en_B^\lambda u_{\lambda q}\exp(i\mathbf{q}.\mathbf{r})}{4\pi\varepsilon_0 A_{BN}}\int_{-\infty}^{+\infty}\int_{-\infty}^{+\infty} dy_1 dx_1 \frac{\exp(-iqx_1)}{(x_1^2+y_1^2+z^2)^{3/2}}\begin{Bmatrix}x_1\\ z\\ y_1\end{Bmatrix}, \tag{2}$$

where A_{BN} is the area of the h-BN unit cell. A direct calculation indicates a vanishing $\Phi_{iT}(\mathbf{r})$ and

$$\Phi_{oT}(\mathbf{r}) = \frac{e n_{B}^{oT} u_{qoT} \exp(i\mathbf{q}.\mathbf{r} - qz)}{2\varepsilon_0 A_{BN}}, \tag{3}$$

$$\Phi_{L}(\mathbf{r}) = -i\frac{e n_{B}^{L} u_{qL} \exp(i\mathbf{q}.\mathbf{r} - qz)}{2\varepsilon_0 A_{BN}}, \tag{4}$$

The vanishing $\Phi_{iT}(\mathbf{r})$ indicates the i-TO phonon has no effective force on the plasmon, where a non-zero Φ indicates coupling between the plasmon and phonon.

Further, we also carried out additional simplified simulation to quantify this coupling phenomenon between the out-plane phonon and the graphene plasmon by the Finite Difference Time Domain (FDTD) method (as discussed in references [9-10]). The simulation results are shown in Supplementary Figure 15. We also present our experimental results in Supplementary Figure 15 for comparison. It shows that the simulation results roughly agree with experimental result. Worth noting that more accurate simulation deserves further investigation.

Supplementary References:

[1] Ju, L. *et al.* Graphene plasmonics for tunable terahertz metamaterials. *Nature Nanotech.* **6**, 630–634 (2011).

[2] Yan, H. *et al.* Tunable infrared plasmonic devices using graphene/insulator stacks. *Nature Nanotech.* **7**, 330–334 (2012).

[3] Brar, V. W., Jang, M. S., Sherrott, M., Lopez, J. J., Atwater, H. A. Highly confined tunable mid-infrared plasmonics in graphene nanoresonators. *Nano Lett.* **13**, 2541–2547 (2013).

[4] Li, Y. *et al.* Graphene plasmon enhanced vibrational sensing of surface-adsorbed layers. *Nano Lett.* **14**, 1573–1577 (2014).

[5] Hu, H. *et al.* Broadly tunable graphene plasmons using an ion-gel top gate with low control voltage. *Nanoscale*. **7**, 19493-19500 (2015).
[6] Liu, F., Cubukcu, E. Tunable omnidirectional strong light-matter interactions mediated by graphene surface plasmons. *Phys. Rev. B* **88**, 115439 (2013).
[7] Shi, Z. *et al.* Gate-dependent pseudospin mixing in graphene/boron nitride moiré superlattices. *Nature Phys.* **10**, 743-747 (2014).
[8] Michel, K. H., Verberck, B. Theory of elastic and piezoelectric effects in two-dimensional hexagonal boron nitride. *Phys. Rev. B* **80**, 224301 (2009).
[9] Brar, V. W. *et al.* Hybrid Surface-Phonon-Plasmon Polariton Modes in Graphene/Monolayer h-BN Heterostructures. *Nano Letter* **14**, 3876-3880 (2014).
[10] Woessner, A.*, et al.* Highly confined low-loss plasmons in graphene–boron nitride heterostructures. *Nature Mater.* **14**, 421-425 (2015).
[11] Gusynin, V. P., Sharapov, S. G., Carbotte, J. P., Magneto-optical conductivity in graphene. J. Phys.: Condens. Matter., **19**, 0262221-02622225 (2007).
[12] Palik, E. D. E. Handbook of optical constants of solids (Academic Press, Orlando, Florida, 1985).
